# Supplementary material for: Myocardin ablation in a cardiac-renal rat model
Source: Sci Rep. 2019 Apr 10;9:5872. doi: 10.1038/s41598-019-42009-z (PMC6458122; doi:10.1038/s41598-019-42009-z)

**Myocardin ablation in a cardiac-renal rat model**

*Anupam Mittal^1&2^,PhD; Santanu Rana^3^,PhD; Rajni Sharma^4^,PhD; Akhilesh Kumar^4^,PhD; Rishikesh Prasad,^4^MSc, Satish K Raut^4^,PhD; Sagartirtha Sarkar^3^,PhD; Uma Nahar Saikia,^5^MD; Ajay Bahl^2^,DM; Perundurai S Dhandapany^1&6*^, PhD; Madhu Khullar^4*^,PhD.*

*^1^ Centre for Cardiovascular Biology and Disease, Institute for Stem Cell Biology and Regenerative Medicine (inStem), Bangalore, India.*

^2^ *Department of Cardiology, Post Graduate Institute of Medical Education and Research, Chandigarh, India.*

*^3^Department of Zoology, University of Calcutta, India.*

*^4^Department of Experimental Medicine and Biotechnology, Post Graduate Institute of Medical Education and Research, Chandigarh, India.*

*^5^Department of Histopathology, Post Graduate Institute of Medical Education and Research, Chandigarh, India.*

*^6^The Knight Cardiovascular Institute, Oregon Health and Science University, Portland, OR, USA Department of Medicine, Oregon Health and Science University, Portland, OR, USA Department of Molecular and Medical Genetics, Oregon Health and Science University, Portland, USA, OR,*

**Short/Running Title: Myocardin ablation in a cardiac-renal rat model**

| **Table S1: Sequences of SYBR Green Chemistry Primers and siRNAs** | | | | |
| --- | --- | --- | --- | --- |
| **Genes** | **Forward Primer** | **Reverse Primer** | **Product Size (bp)** | **T_m_**  **(°C)** |
| **Rno-Myocardin** | 5’AGATCCCGGC  CGCTTCCCTTT’3 | 5’GGTCGGGAAT  CTGGGAGAGG’3 |  | 58 |
| **Rno-CTGF** | 5’GAGTCGTCTC  TGCATGGTCA‘3 | 5’CCACAGAACT  TAGCCCGGTA‘3 | 156 | 58 |
| **Rno-FGF-β** | 5’CGGTACCTGG  CTATGAAGGA‘3 | 5’CCGTTTTGGA  TCCGAGTTTA‘3 | 178 | 58 |
| **Rno-TGF-β** | 5’TGCTTCAGCTC  CACAGAGAA‘3 | 5’TGGTTGTAGA  GGGCAAGGAC‘3 | 182 | 58 |
| **Rno-Col-1a** | 5’TGCTGCCTTT  TCTGTTCCTT‘3 | 5’AAGGTGCTGG  GTAGGGAAGT‘3 | 179 | 58 |
| **Rno-Col-3a** | 5’CATCTTTTCCA  GGAGGTCCA’3 | 5’GTCCACGAGGT  GACAAAGGT‘3 | 189 | 58 |
| **Rno-Col-4a** | 5’GCCAAGTGTG  CATGAGAAGA‘3 | 5’AGCGGGGTGT  GTTAGTTACG‘3 | 202 | 58 |
| **Rno-ANP** | 5’ATTTCAAGAACC  TGCTAGACC’3 | 5’TTTTCAAGAG  GGCAGATCTAT’3 | 222 | 58 |
| **Rno-β-MHC** | 5’CCTCGCAATAT  CAAGGGAAA’3 | 5’TACAGGTGCAT  CAGCTCCAG’3 | 198 | 58 |
| **Hsa/Rno-GAPDH** | 5’ACAGCAACAG  GGTGGTGGAC‘3 | 5’TTTGAGGGTG  CAGCGAACTT‘3 | 252 | 58 |
| **Rno-Myocardin siRNA 1** | 5’GGUCAAACCCA  UGUACUCUTT‘3 | 5’AGAGUACAUGGG  UUUGACCTG‘3 |  |  |

Atrial Natriuretic factor (ANP), Beta-Myosin heavy chain (β-MHC), Collagen (Col) 1a, Col 3a, Col 4a, Transforming growth factor-β (TGF-β), Connective tissue growth factor (CTGF) and Fibroblast growth factor (FGF)- β

| **Table S2: Source and Working Concentration information of antibodies** | | | |
| --- | --- | --- | --- |
| **S.No.** | **Antibody** | **Protein MW (kDa)** | **Dilution Factor**  **(DF)** |
| **1** | **Myocardin (Sigma; #SAB4200539)** | 105 kDa | 1:1000 |
| **2** | **ANP (Thermo Pierce; sc-18811)** | 17 kDa | 1:750 |
| **3** | **β-MHC (Santacruz; sc-168678)** | 190 kDa | 1:1000 |
| **4** | **GAPDH (Sigma; G9545)** | 36 kDa | 1:1000 |
| **5** | **CTGF (Santacruz; sc-365970)** | 38 kDa | 1:700 |
| **6** | **FGF (Santacruz; sc-1390)** | 19 kDa | 1:1000 |
| **7** | **Anti-mouse IgG-HRP**  **(Santacruz; A9919)** |  | 1:10000 |
| **8** | **Anti-goat IgG-HRP**  **(Santacruz; A5420)** |  | 1:10000 |
| **9** | **Mouse anti-rabbit IgG-HRP**  **(Santacruz; sc-2357)** |  | 1:10000 |

**
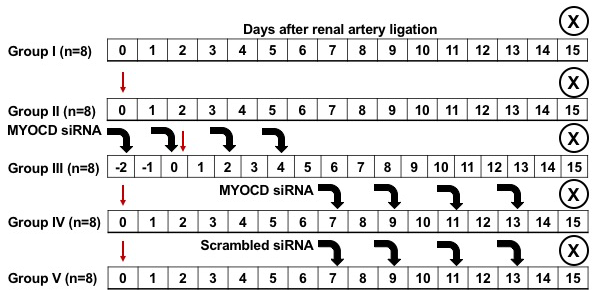
Supplementary Figure 1: Experimental Design of siRNA treatment in RAL group:** 20 weeks old wistar rats were randomized in five groups, assigned to undergo ligation of right renal artery and SHAM procedure. Group I: SHAM operated rats (Control group), Group II: Ligated rats (RAL group), Group III: Myocardin (MYOCD) siRNA introduced in ligated group after ligation and initiation of cardiac remodelling (Post-Ligation group), Group IV: MYOCD siRNA introduced in ligated group before ligation and initiation of cardiac remodelling (Pre-Ligation group). Group V: Scrambled siRNA introduced in ligated group. Red arrow shows the day of ligation. Black coloured bold arrow shows days on which MYOCD siRNA/Scrambled negative siRNA was introduced and Encircled cross shows day of sacrificing rats.

**
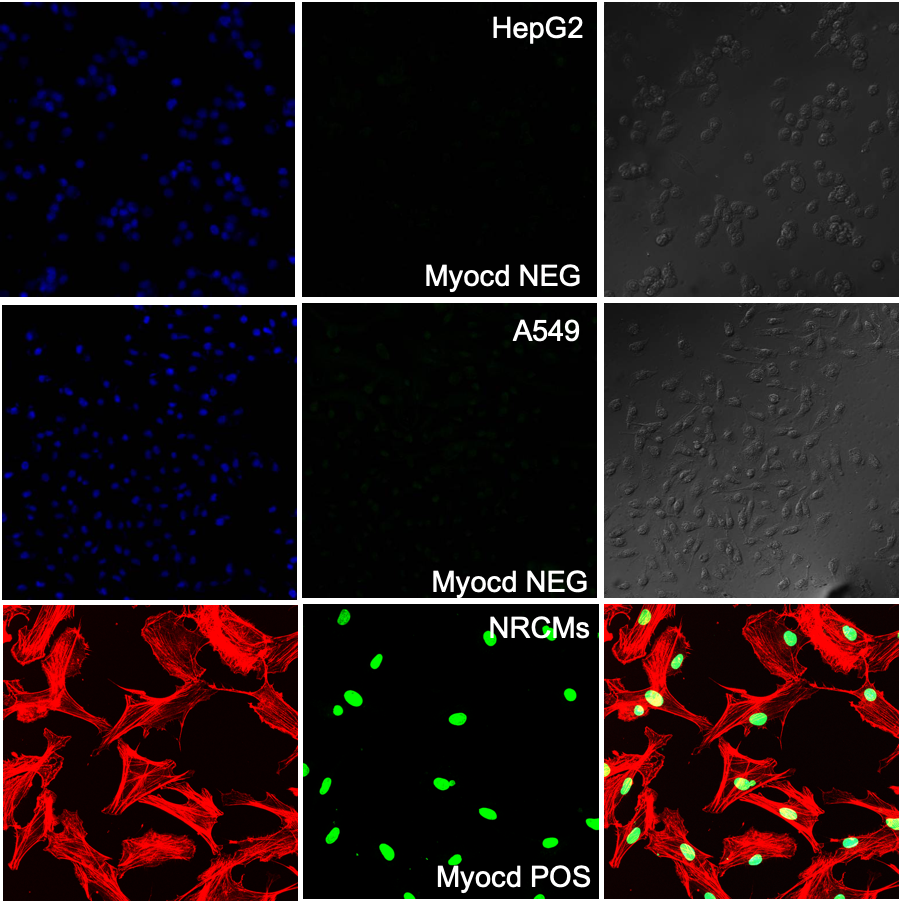
**

**Supplementary Figure 2: Myocardin (MYOCD) immunostaining in various cell lines to confirm the specificity of the antibody.** Upper Panel: Immunocytochemistry done in HepG2 hepatocarcinoma cells (Anti-MYOCD dilution: 1:200) shows negative expression of MYOCD. Middle Panel: A549 cells stained with same dilution of MYOCD shows very faint (nearly negatively) stained cells. Lower Panel: Neonatal rat cardiomyocytes (NRVCs) stained with 1:200 anti-MYOCD show good MYOCD expression. MYOCD antibody was used even at lower dilution of 1:100 in HepG2 and A549 but showed similar results (data not shown).

**
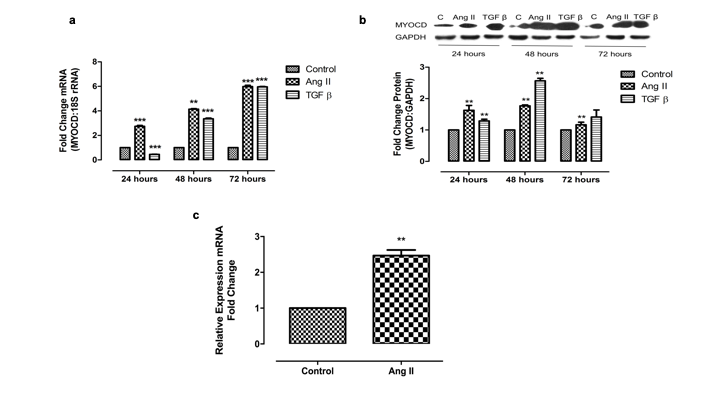
**

**Supplementary Figure 3: Myocardin (MYOCD) expression in Ang-II and TGF β treated H9c2 cardiomyoctes and cardiac fibroblast cells.** H9c2 cardiomyocytes and cardiac fibroblast were incubated with 1µM of Ang II for 24 hours to induce hypertrophy and fibrosis **a)** MYOCD mRNA expression was determined by the qRT-PCR in H9c2 cardiomyocytes and **b)** Representative blot showing the expression of MYOCD in time dependent manner in H9c2 cardiomyocytes **c)** MYOCD mRNA determination in Ang-II treated cardiac fibroblasts. GAPDH was used for normalization of the qRT-PCR and protein expression analysis. Data given is Mean ± SEM. *p<0.05, **p<0.01, ***p<0.001 Control vs Ang II; Control vs TGF β.

**
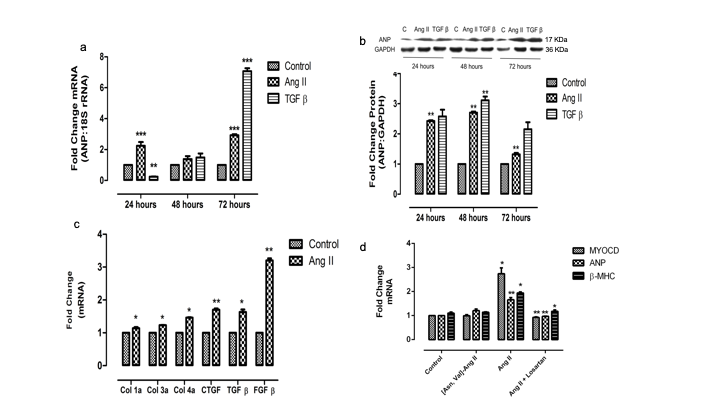
**

**Supplementary Figure 4. Effect of** **MYOCD overexpression on Ang II induced expression of hypertrophy and fibrosis genes in H9c2 cardiomyocytes and fibroblasts respectively a)** Quantitative mRNA expression of Atrial Natriuretic factor (ANP) in Ang-II (1µM) and TGF β (7.5 ng/ml) treated H9c2 cells as determined by qRT-PCR. **b)** Representative western blot for ANP and GAPDH proteins in Ang-II and TGF-β treated H9c2 cells. **c)** Quantitative mRNA expression of Collagen (Col) 1a, Col3a, Col4a, CTGF, TGF-β and FGF-β (fibrotic marker gene) in Ang II treated cardiac fibroblasts as determined by qRT-PCR. **d)** Angiotensin II type I receptor antagonist, Losartan treatment in Ang II treated H9c2 confirmed that increase in MYOCD and hypertrophy genes (ANP and β-MHC) is mediated by Ang II. Normalization of total RNA and protein was done by using GAPDH as an internal control. Data given is Mean ± SEM of triplicates; *p<0.05; **p<0.01; ***p<0.001 Control vs Ang II or Ang II vs Ang II + Losartan and Control vs TGF


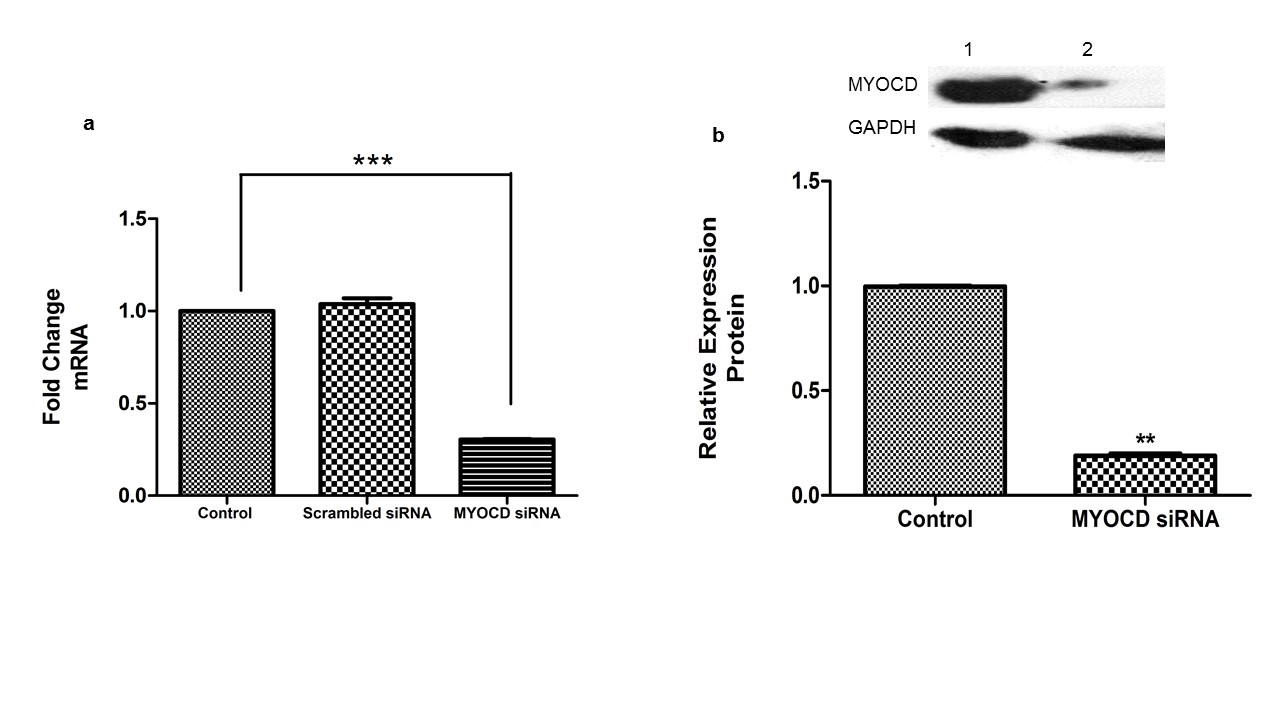


**Supplementary Figure 5: Expression of Myocardin (MYOCD) after transfection with MYOCD specific siRNA into the cardiomyocytes. a)** MYOCD mRNA expression after transfection with MYOCD siRNA. **b)** Representative western blot and quantitative results of the MYOCD and GAPDH protein expression. Lane1: Cells with only vector, Lane 2: Cells transfected with MYOCD siRNA. Normalization was done using GAPDH as internal control. Data shown are results from three independent experiments run in triplicate. Data given is Mean ± SEM; **p<0.01, ***p<0.001 Control vs MYOCD siRNA.

**Supplementary Information**

Figure 1: Western Blot showing MYOCD expression in all control (n=10) and DCM patient biopsies (n=15) other than which has been shown as representative images in the main figure 1b


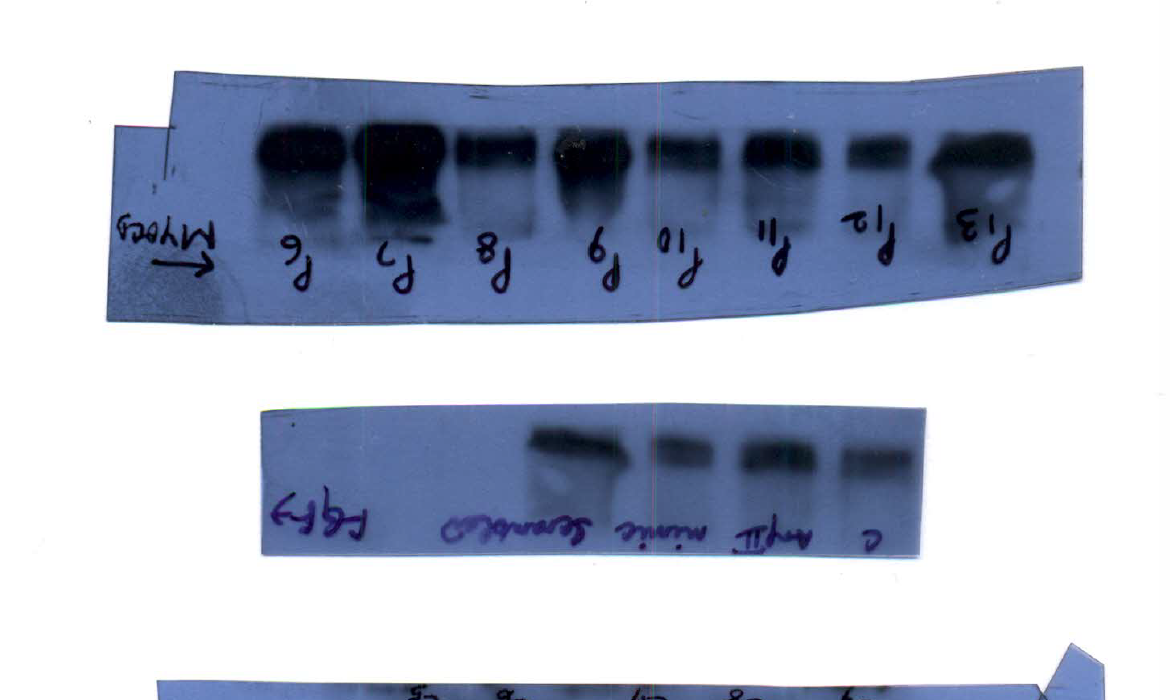


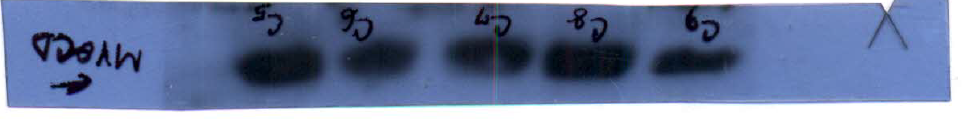


Figure 2: Raw western blots showing ANP expression after Ang II and TGF-β in time dependent manner in H9c2 cells (Sequence from left to right 1. Control 24 hrs, 2. Ang II 24 hrs, 3. TGF- β 24 hrs, 4. Control 48 hrs, 5. Ang II 48 hrs, 6. TGF- β 48 hrs, 7. Control 72 hrs, 8. Ang II 72 hrs, 3. TGF- β 72 hrs)


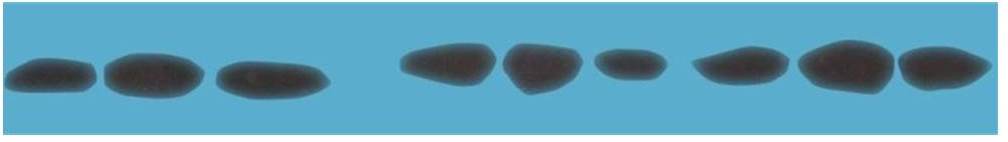


GAPDH

ANP


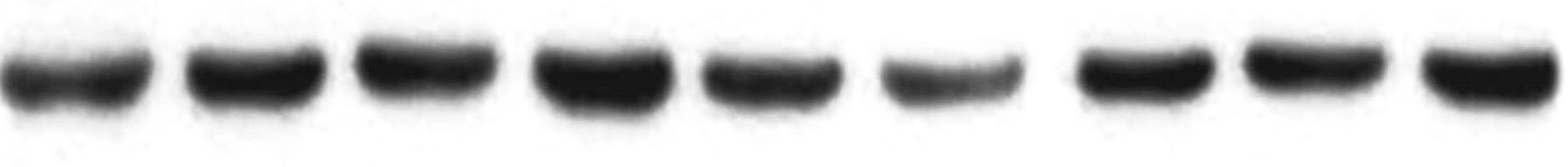


Figure 3: Unprocessed western blot showing ANP and MYOCD protein expression after treatment with MYOCD siRNA in Ang II treated H9c2 cells (Sequence from Left to right: 1. Control H9c2, 2. Ang II, 3. Ang II + MYOCD siRNA1, 4. Ang II + MYOCD siRNA 2). siRNA2 data is not shown in Final figure


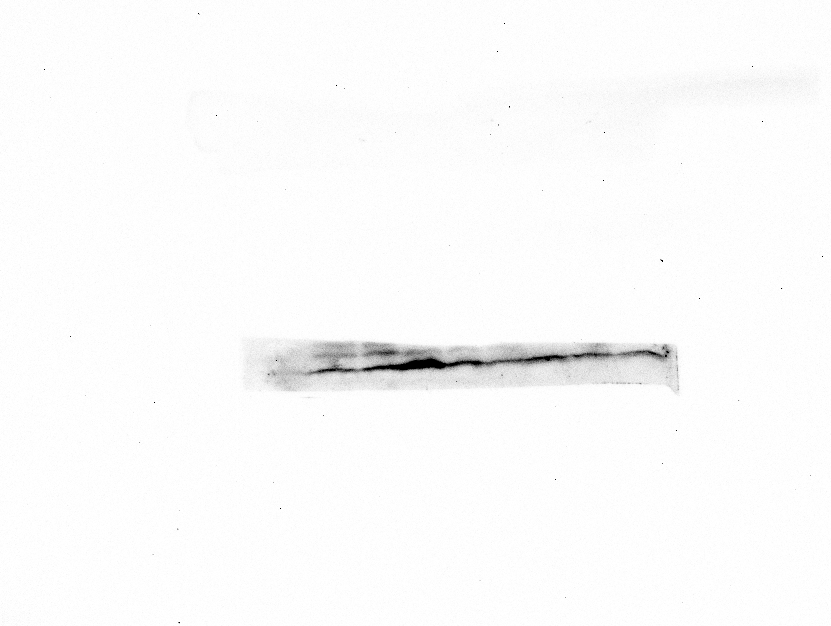


ANP


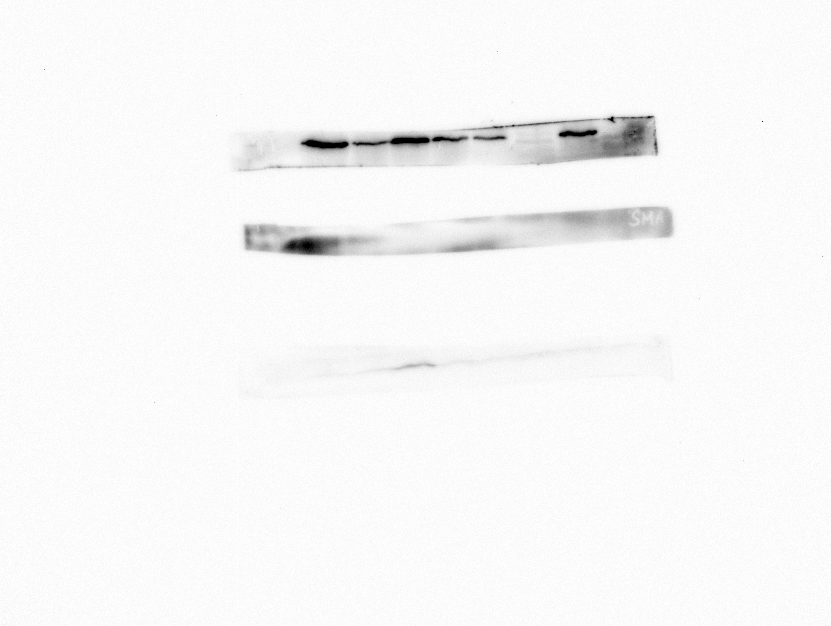


MYOCD


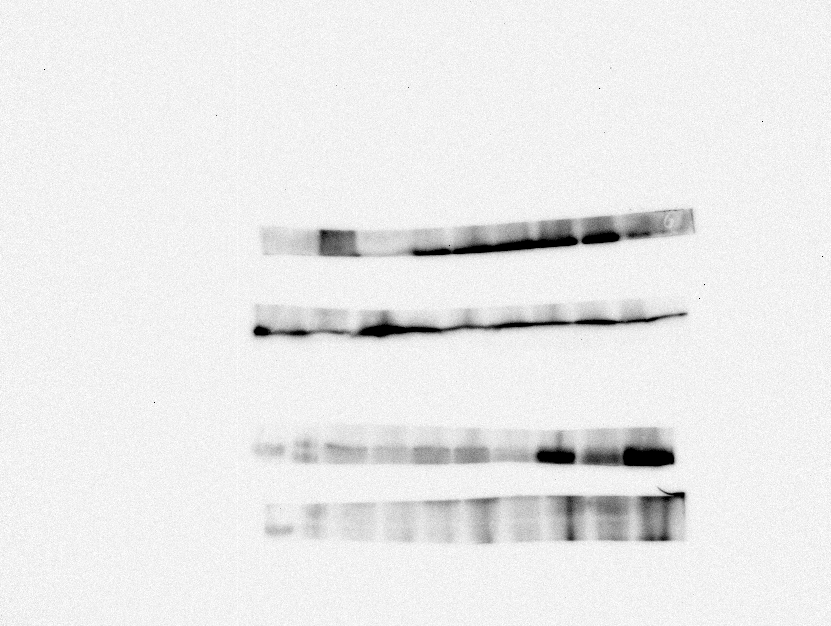


GAPDH

Figure 4: Unprocessed western blot for MYOCD, ANP and GAPDH proteins in MYOCD siRNA treated RAL rats (before and after ligation). Sequence from Left to right is: 1. Control rat 2. Renal artery ligated rat 3. Renal artery ligation + MYOCD silencing Pre-ligation 4. Renal artery ligation + MYOCD silencing Post-ligation 5. Renal artery ligation + Scrambled (negative siRNA)


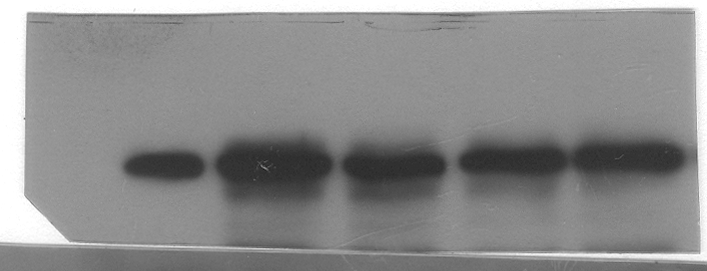


ANP


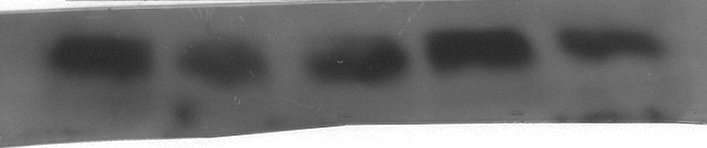


MYOCD


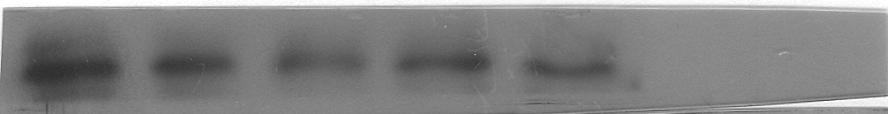


βMHC


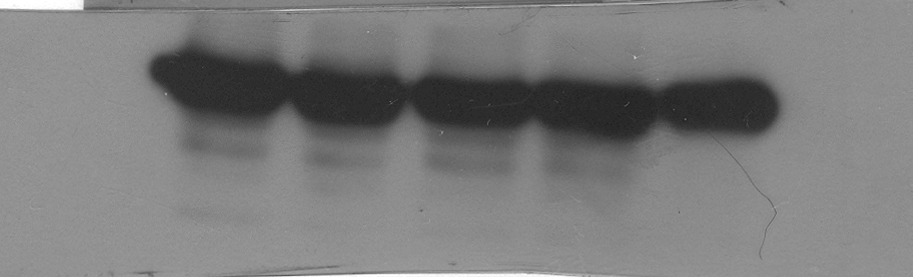


GAPDH

Figure 5: Unprocessed western blot for FGF, CTGF, MYOCD and GAPDH proteins in MYOCD siRNA treated RAL rats (before and after ligation). Sequence from Left to right is: 1. Control rat 2. Renal artery ligated 3. Renal artery ligation + Scrambled (negative siRNA) 4. Renal artery ligation + MYOCD silencing Pre-ligation 5. Renal artery ligation + MYOCD silencing Post-ligation


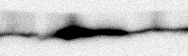


FGF

CTGF


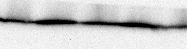


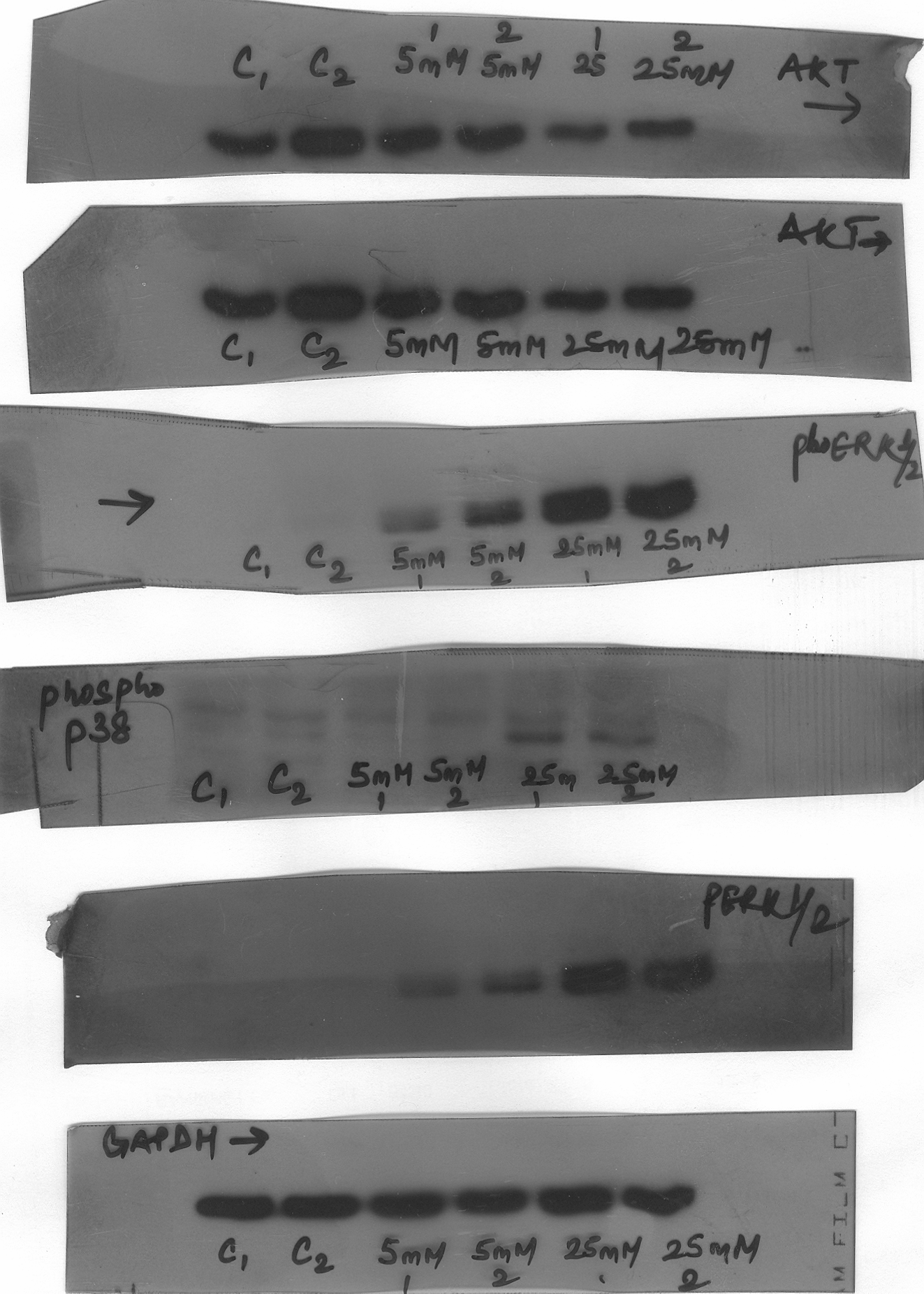


MYOCD

GAPDH


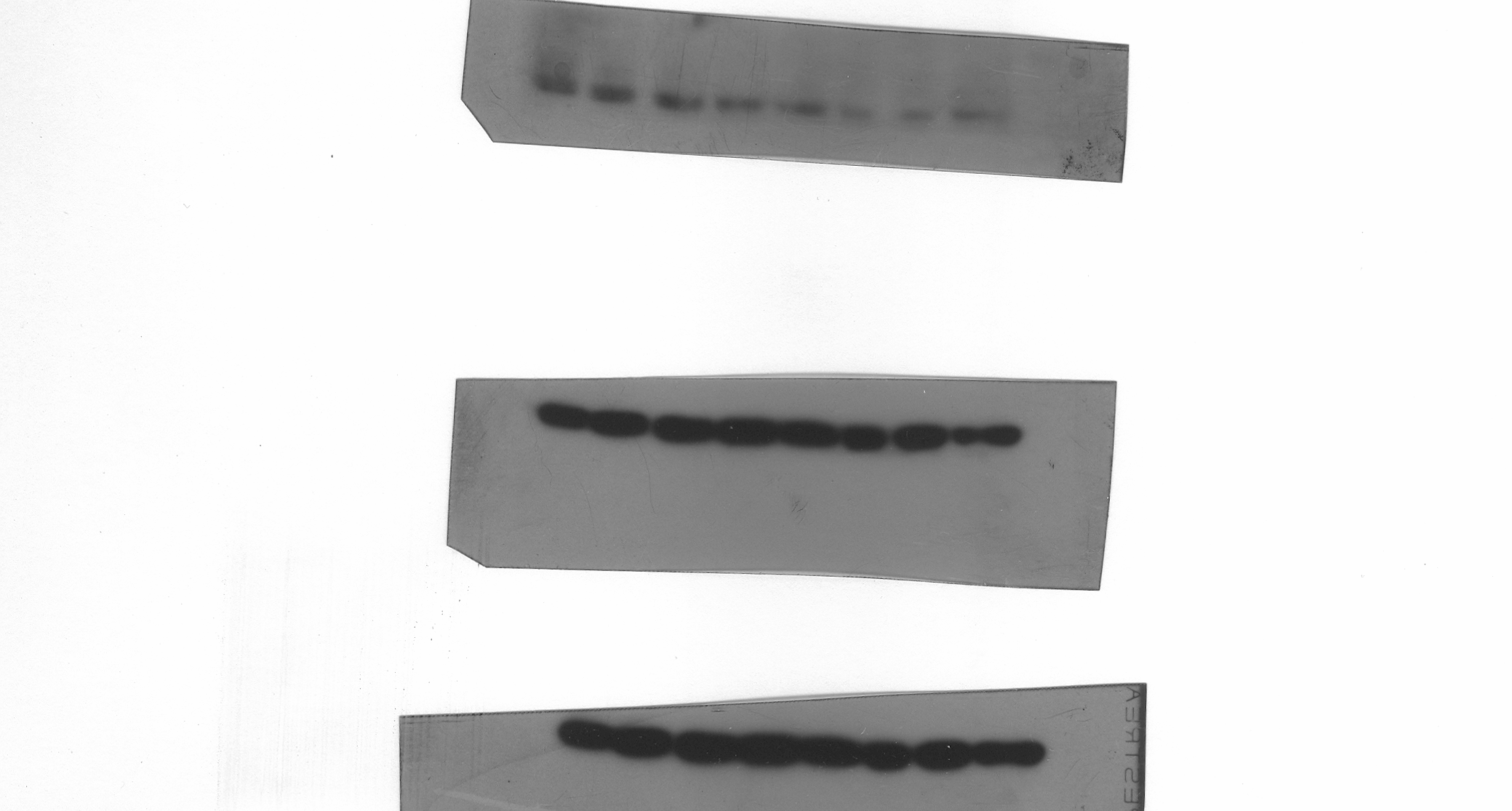


Figure 6a-d: H&E staining at lower magnification a) Control rat b) Renal artery ligated c) Renal artery ligation + MYOCD silencing Pre-ligation d) Renal artery ligation + MYOCD silencing Post-ligation. 6 e-h: MT staining at lower magnification e) Control rat f) Renal artery ligated g) Renal artery ligation + MYOCD silencing Pre-ligation h) Renal artery ligation + MYOCD silencing Post-ligation.


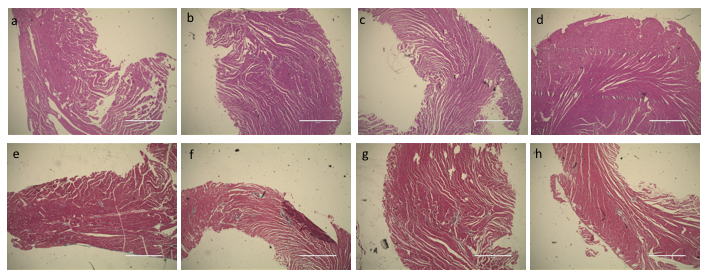

Supplement: Supplementary file 1 — Dataset 1 [file 41598_2019_42009_MOESM1_ESM.doc]
